# Event and Comment

**Published:** 1913-10-15

**Authors:** 


### DENTAL REGISTER.

**Vol. LXVII. October 15, 1913. No. 10**

### EVENT AND COMMENT.

GERMICIDAL TOOTH POWDERS. This is the day of germ killing remedies. Few are the diseases known today that have not had their causal factors traced to the bacterial activity, consequently the germicide and antiseptic hold the first place in all efforts to correct and prevent the spread of disease in the human body. Because of the discovery that dental caries was a microbic disease efforts have been made to discover a microbic cause for peridentitis, with so far no conclusive evidence to substantiate the theory. At any rate we know no better way of treating this malady than by the disinfectant, surgical, and therapeutic methods. Formerly our tooth powders were made up of cleansing or abrasive materials with sugar, soap and flavoring materials to increase the detergent effects and make them palatable. Today the mechanical and chemical abrasives have to some extent given way to the germicidal and antiseptic ingredients. In spite of the demonstrated fact that continued sterilization of the mouth is impracticable with the most potent chemical germicides that are admissable in the mouth, our manufacturers of tooth dentifrices are making strong claims for their various preparations on account of their germicidal properties. Patents on supposed valuable formulae have been taken out to monopolize the market for some supposedly valuable discovery in the nature of a tooth dentifrice. In some cases it is claimed by the promoters that their dentifrices will stop caries, prevent tartar and cure pyorrhea alveolaris. The Council of Pharmacy and Chemistry of the American

Medical Association recently analized a widely advertised dentifrice which claimed to contain an ingredient which would when dissolved in the oral secretions liberate a powerful germicidal element in safe but sufficient quantities to overcome all putrefactive activity in the mouth. The analysis showed an insignificant and wholly inadequate supply of the ingredient to be produced under the most favorable conditions, and it also reports that this element was not produced by the reagent claimed by the manufacturers, which was practically insoluable in the mouth under anything like normal conditions.

Is it not high time the dental profession was demanding non-secret medical and therapeutic remedies? How long are we to be pauperized by an indifference to the higher demands of definite and accurate scientific knowledge of the remedies we use? It is so easy to buy and use proprietary remedies that the great mass of the profession never raise a question as to the ethics of such matters. Most of us insist on knowing exactly what the ingredients are with their proportions of cocain solutions, which we are using because we fear we may need this information for justification in case of a possible accident. But we seem utterly indifferent as to the composition of the dentifrice we recommend to our patients, who use them with unrestrained confidence because they believe we know exactly what we are prescribing, and that we have their best interests in view. We believe it is more important that the dentifrice we place in the hands of our patients shall be a safe one than that the cocain solution shall be definitely known, because we never place the cocain solution in the hands of our patients for unlimited use without constant professional surveilance, while we do place in the hands of our patients a powerful chemical compound for continued use perhaps for years with no intimation that it may do any injury. We have seen many cases of detrimental and irreparable injury resulting from the excessive use of tooth dentifrices. Every dentist who closely observes his cases will find these results. The campaign for clean teeth that is sweeping our land is making a tremendous demand for tooth toilet facilities, but we see no indications anywhere that the profession is making any move to supply the people with a safe and reliable dentifrice, or that the profession itself is seriously considering the problem of efficient and wholesome mouth hygiene. We seem to be inflated with the idea of prevention, but are doing little in devising safe and practicable means for securing it. Let's throw overboard all secret nostrums and get down to the business of prescribing proper remedial agents for diseased oral conditions and suitable sanitary procedures for the oral toilet. We shall need to become ourselves intelligent on the entire subject before we can prescribe for or instruct our patients. Can't we have a Council of Pharmacy in our newly organized National Association to protect and educate us?

